# Supplementary material for: A highly active Ni(II)-triadamantylphosphine catalyst for ultrahigh-molecular-weight polyethylene synthesis
Source: Nat Commun. 2019 Jan 25;10:438. doi: 10.1038/s41467-019-08309-8 (PMC6347634; doi:10.1038/s41467-019-08309-8)
Supplement: Supplementary file 1 — Supplementary Information [file 41467_2019_8309_MOESM1_ESM.pdf]

Supplementary Information

A Highly Active Ni(II)-Triadamantylphosphine Catalyst for Ultrahigh-Molecular-Weight Polyethylene Synthesis

Andrew L. Kocen, Maurice Brookhart, and Olafs Daugulis

Welch Center for Excellence in Polymer Chemistry, Department of Chemistry, University of Houston, Houston, Texas 77024-5003, USA.

Table of Contents

	Page
**Supplementary Notes**	**S-2**
**Supplementary Methods**	**S-4**
**Supplementary Tables**	**S-13**
**Supplementary Figures**	**S-17**
**Supplementary References**	**S-24**

Supplementary Notes

Reactions were performed using standard glassware or were run in 2-dram vials with PTFE/Liner screw caps. The ^{1}H, ^{13}C, and ^{31}P spectra were recorded on JEOL EC-400, JEOL EC-500, or JEOL EC-600 spectrometers using the residual solvent peak as a reference. Comonomer incorporation for 1-hexene and 1-octadecene was calculated using inverse gated decoupling with 0.05 M $Cr(acac)_3$ as a relaxation agent in C_6D_5Br at 140 °C using ^{13}C NMR. Comonomer incorporation for tert-butyldimethyl(dec-9-en-1-yloxy)silane-ethylene copolymer was calculated using ^{1}H NMR in C_6D_5Br at 130 °C. Elemental analyses were performed by Atlantic Microlabs Inc. (Norcross, GA, USA). All procedures were performed under a nitrogen atmosphere unless otherwise noted. Reagents and starting materials were obtained from commercial sources and used without further purification unless otherwise noted. Ad_3P,[1] tert-butyldimethyl(dec-9-en-1-yloxy)silane,[2] and $[tBuP_3NiBr_3][tBuP_3H]$[3] were made according to the known procedures. NaBArF is sodium tetrakis[3,5-bis(trifluoromethyl)phenyl]borate. Polymerization reactions were carried out in a Parr 450 mL mini reactor or a Parr 1L stand reactor with Matheson purity ethylene from Matheson Gas. PMAO-IP was purchased as a 7 wt% solution in toluene from AkzoNobel.

X-Ray measurements were made with a Bruker DUO platform diffractometer equipped with a 4K CCD APEX II detector and an Incoatec 30 Watt Cu microsource with compact multilayer optics. Data was collected using a narrow-frame algorithm with scan widths of 0.50\% in omega and an exposure time of 25 s/frame at 123 K. The data were integrated using the Bruker-Nonius SAINT program, with the intensities corrected for Lorentz factor, polarization, air absorption, and absorption due to variation in the path length through the detector faceplate. The data were scaled, and an absorption correction was applied using SADABS. Redundant reflections were averaged. The structure was solved by using the program SHELXT and refined using SHELXL.

DSC measurements were collected on a TA instruments Q2000 using a heat-cool-heat method with a nitrogen flow of 50 mL/min. The sample was heated at 10 °C/min from 40 °C to 160 °C then cooled from 160 °C to 0 °C at 10 °C/min. The sample was then heated from 0 °C to 160 °C at 10 °C/min. All reported melting temperatures come from the second heating cycle.

GPC analysis was performed on a Malvern HT-GPC using triple detection and a polystyrene standard to determine absolute molecular weight. Three mixed bed columns were used with a 1,2,4-trichlorobenzene flow of 1.0 mL/min at 150 °C. All samples were dissolved in 1,2,4-trichlorobenzene at 160 °C. Samples were heated for at least two hours to dissolve, but heating was kept under six hours to avoid chain degradation from occurring.

Supplementary Methods

Synthesis of Catalyst 6

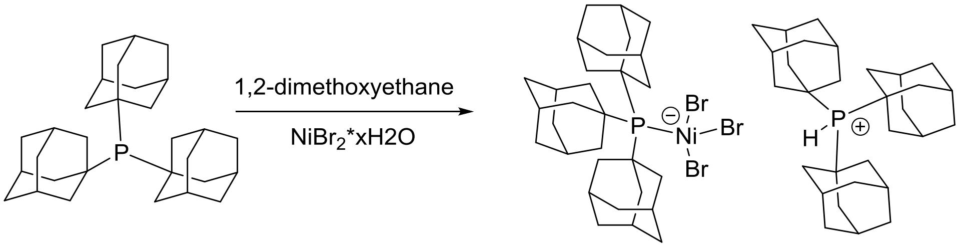

6, 48 %

In a 100 mL flame dried Schlenk flask, nickel bromide hydrate (273 mg, 1 mmol) and tri(1-adamantyl)phosphine (436 mg, 1 mmol) were combined under nitrogen. The flask was evacuated and refilled with nitrogen three times. Dimethoxyethane (20 mL) was added and the mixture was stirred for 19 hours. Diethyl ether (80 mL) was added to precipitate product as a green solid. The supernatant liquid was removed via cannula and the remaining solid was dried under vacuum. The resulting green solid was dissolved in dichloromethane and filtered through Celite® to remove excess nickel bromide. The green solution was concentrated in vacuo. The product was then redissolved in chlorobenzene and diethyl ether was added at -30 °C to recrystallize **6**. The product was filtered in air and washed with diethyl ether. The resulting green solid was dried under vacuum to give **6** (281 mg, 0.24 mmol, 48%) as a green solid. X-Ray quality crystals were grown by vapor diffusion of diethyl ether into a chlorobenzene solution of **6** at room temperature. Complex **6** decomposes slowly in solution but is stable as a solid in air for at least 6 months.

Anal. Calcd. for $C_{60}H_{91}Br_3NiP_2$: C, 61.45, H, 7.82; Found: C, 61.47 H, 7.71. – No additional purification was performed before elemental analysis was done.

^{1}H NMR (600 MHz, CD_2Cl_2, ppm): δ 5.98 (br apparent s, 18H), 3.29 (br apparent s, 9H), 2.97 (d, J = 10.3 Hz, 9H), 2.47 (d, J = 9.6 Hz, 9H).

The ^{1}H NMR spectra show only the spectrum of the cation as broad peaks, shifted from their expected range. As the compound decomposes in solution the signals shift to the expected values for the free cation. The spectrum shown was taken approximately 30 minutes after dissolution of **6** in NMR solvent. No ^{13}C or ^{31}P signals were observed as **6** is paramagnetic.

Synthesis of Catalyst 8

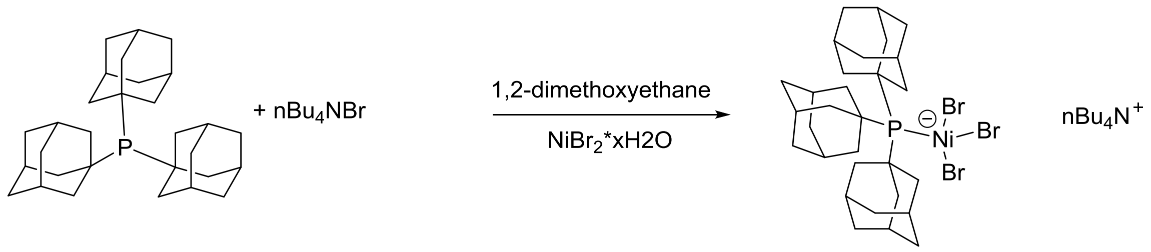

8, 82 %

In a 2 dram vial tris(1-adamantyl)phosphine (44 mg, 0.1 mmol), tetrabutylammounium bromide (32 mg, 0.1 mmol), and nickel bromide hydrate (27 mg, 0.1 mmol) were stirred in 1,2-dimethoxyethane (2 mL) under nitrogen for 16 hours. The solution turned green and pentane (5 mL) was added. A green solid percipitated out of solution and was filtered and wased with pentane (3 x 5 mL). The solid was dried under a strong vacuum to give **8** (80 mg, 0.082 mmol, 82%) as a green solid.

^{1}H NMR (600 MHz, CD_2Cl_2, ppm): δ 10.12 (br apparent s, 8H), 6.05 (br apparent s, 8H), 4.03 (br apparent s, 8H), 2.04 (br apparent s, 12H).

The ^{1}H NMR spectra show only the spectrum of the cation as broad peaks, shifted from their expected range. As the compound decomposes in solution the signals shift to the expected values for the free cation. The spectrum shown was taken approximately 5 minutes after dissolution of **8** in NMR solvent. Signals of the free ligand appear as the catalyst decomposes. The signals for Bu_4N^+ fragment shift towards those of free cation. No ^{13}C or ^{31}P signals were observed as **8** is paramagnetic.

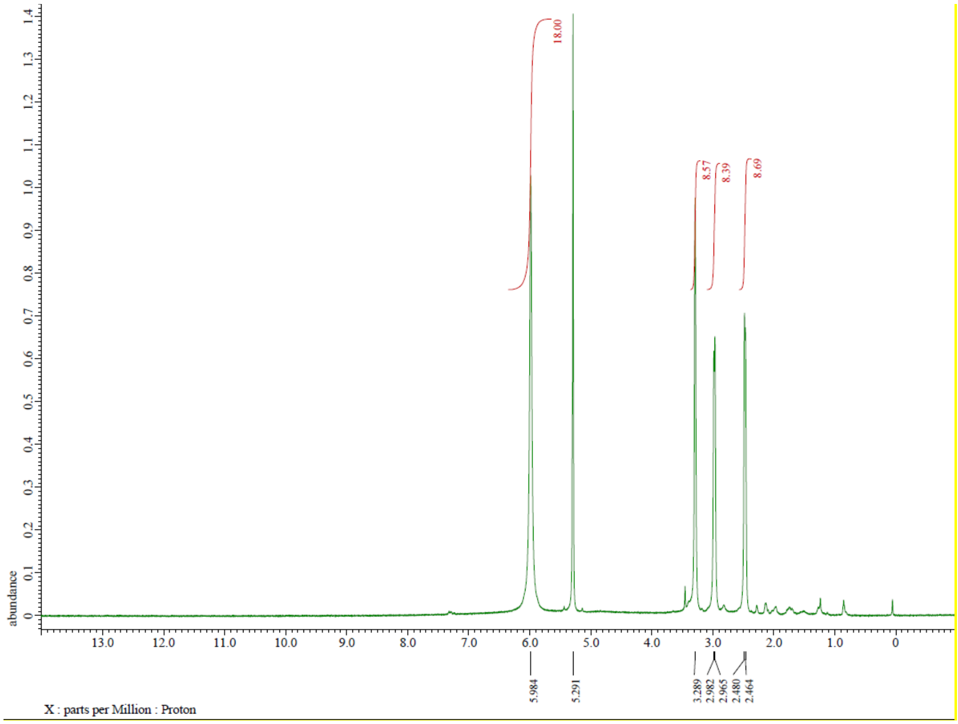

Supplementary Figure 1: ^{1}H NMR spectrum of **6**

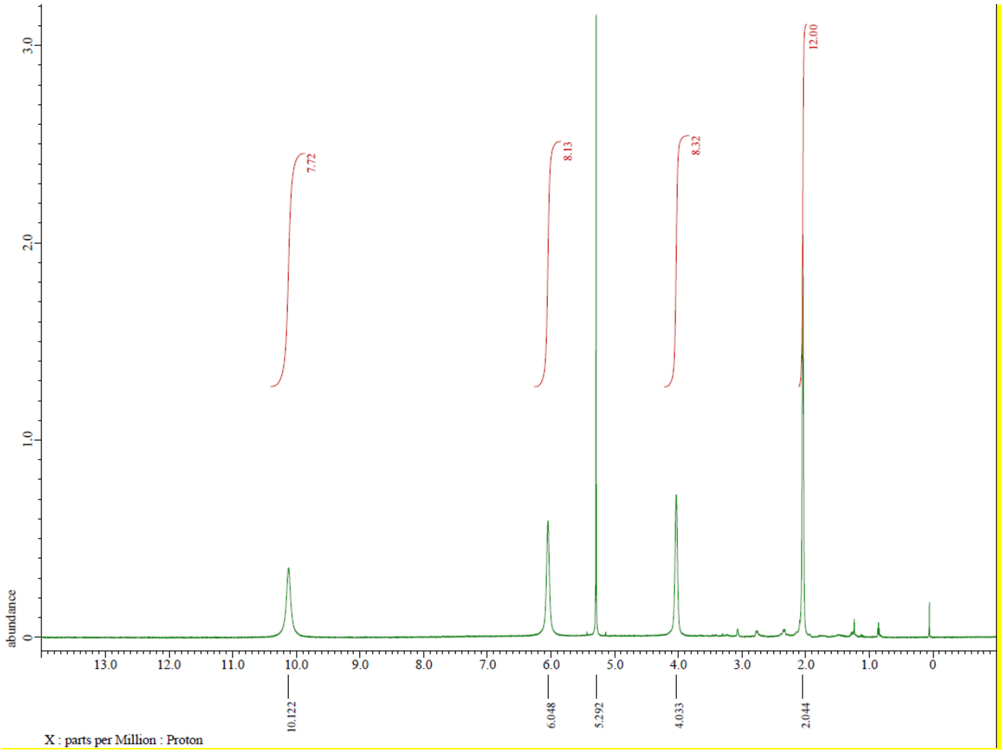

Supplementary Figure 2: ^{1}H NMR spectrum of **8**

Catalyst Stability Experiments

A 50 mL oven dried Schlenk flask was cooled to 0 °C under nitrogen. Toluene (25 mL) and PMAO-IP (0.39 mL, 1750 eq) were added via syringe under nitrogen. Ethylene (1.12 mL, 100 eq) was added via syringe directly into the solution followed by **6** (0.5 µmol, 1 eq in 0.5 mL CH_2Cl_2) and the mixture was stirred for the off time. After the off-time had elapsed, ethylene was purged directly into solution with a bubbler attached to the flask to allow for excess ethylene to vent off. For Table 3, entry 1, ethylene was purged directly into solution prior to catalyst addition (in place of addition of ethylene via syringe). After 20 minutes of stirring ethylene was removed and the flask was opened to air. The reaction mixture was quenched with a 1:1 10% HCl(aq)/MeOH solution (25 mL) and filtered. The polymer was washed with acetone and dried overnight in a vacuum oven.

Supplementary Table 1: Decomposition study

Catalyst **6** → Polyethylene
1) Toluene, PMAO-IP, 100 eq C_2H_4, 0°, X min
2) C_2H_4 (1 atm), 20 min

#	Off-time (min)	Yield (mg)	% dead	M_w x 10^{-6}	M_n x 10^{-6}	Đ
1	0	770	0	1.61	1.15	1.40
2	10	565	27%	1.23	0.94	1.31
3	30	360	54%	1.80	1.28	1.41
4	60	110	86%	1.60	1.10	1.46

Standard conditions: catalyst **6** 0.5 µmol, PMAO-IP 1750 eq, toluene 25 mL, 100 equiv ethylene added via syringe into solution; wait X minutes, then bubble ethylene directly into solution. Molecular weight determined by GPC in 1,2,4-trichlorobenzene.

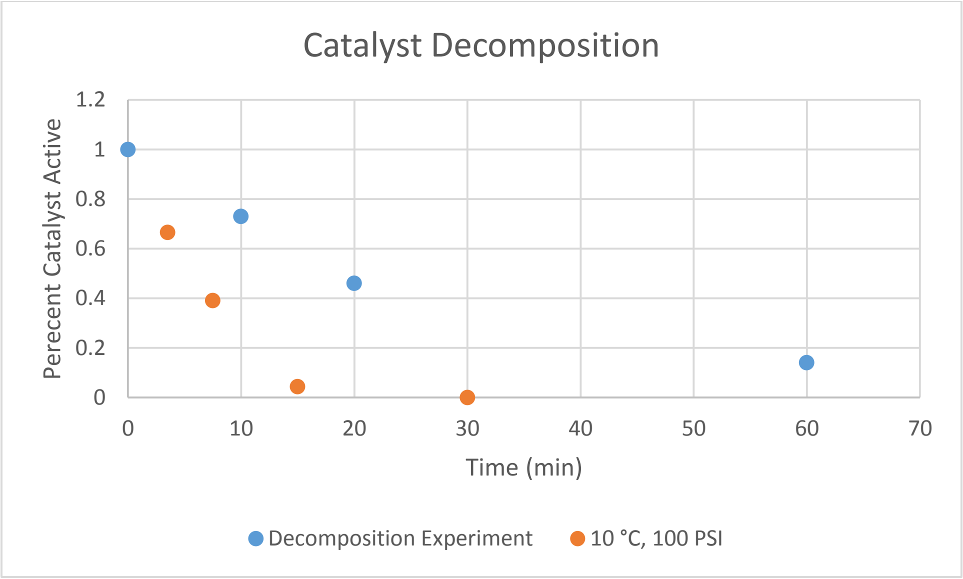

Supplementary Figure 3: Comparison of decomposition at 100 PSI, 10 °C (from Supplementary Table 3) with data from Supplementary Table 1.

Synthesis of Palladium Catalyst 9

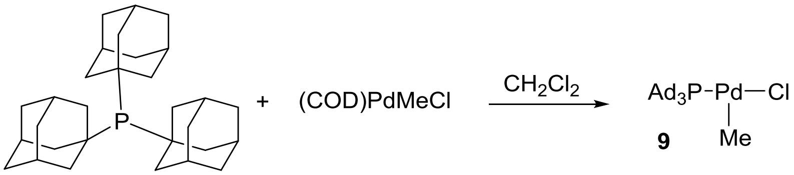

To a 2-dram vial equipped with a PTFE septa, tris(1-adamantyl)phospine (86 mg, 0.2 mmol) and (COD)PdMeCl (53 mg, 0.2 mmol) were added in a nitrogen filled glovebox. Outside of the glovebox, methylene chloride (2 mL) was added via syringe and the solution was stirred for 30 minutes. The reaction was poured into hexanes (20 mL) and cooled to -30 °C to crystallize. The solid was filtered, washed with hexanes (3 x 10 mL), and dried under vacuum to give **9** (56 mg, 0.094 mmol, 47%) as a yellow solid.

^{1}H NMR (400 MHz, $CDCl_3$, ppm): δ 2.35 (apparent br s, 15H), 2.02 (apparent br s, 9H), 1.69-1.81 (m, 18H).

^{13}C NMR (101 MHz, $CDCl_3$, ppm): δ 47.8 (d, J = 7.7 Hz), 42.1 (apparent br s), 36.4, 29.0 (d, J = 7.7 Hz), -2.9.

^{31}P NMR (162 MHz, $CDCl_3$, ppm): 53.6.

Attempted Polymerization of Ethylene Using 9

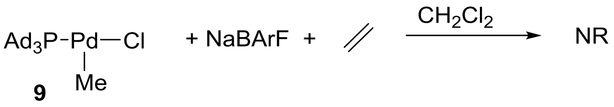

In a 100 mL flame-dried Schlenk flask, **9** (5.9 mg, 0.01 mmol) and NaBArF (8.9 mg, 0.01 mmol) were combined in a nitrogen filled glovebox. The flask was sealed with a septum. The flask was equipped with a bubbler and flushed with ethylene using a syringe connected to ethylene tank. Methylene chloride (20 mL) was added, and solution was stirred with the ethylene bubbled through the solution. After twenty minutes the ethylene tank was disconnected and nitrogen was purged through the solution to remove ethylene. An aliquot of solution was analyzed by ^{1}H NMR for the presence of oligomers or polymer but none were observed.

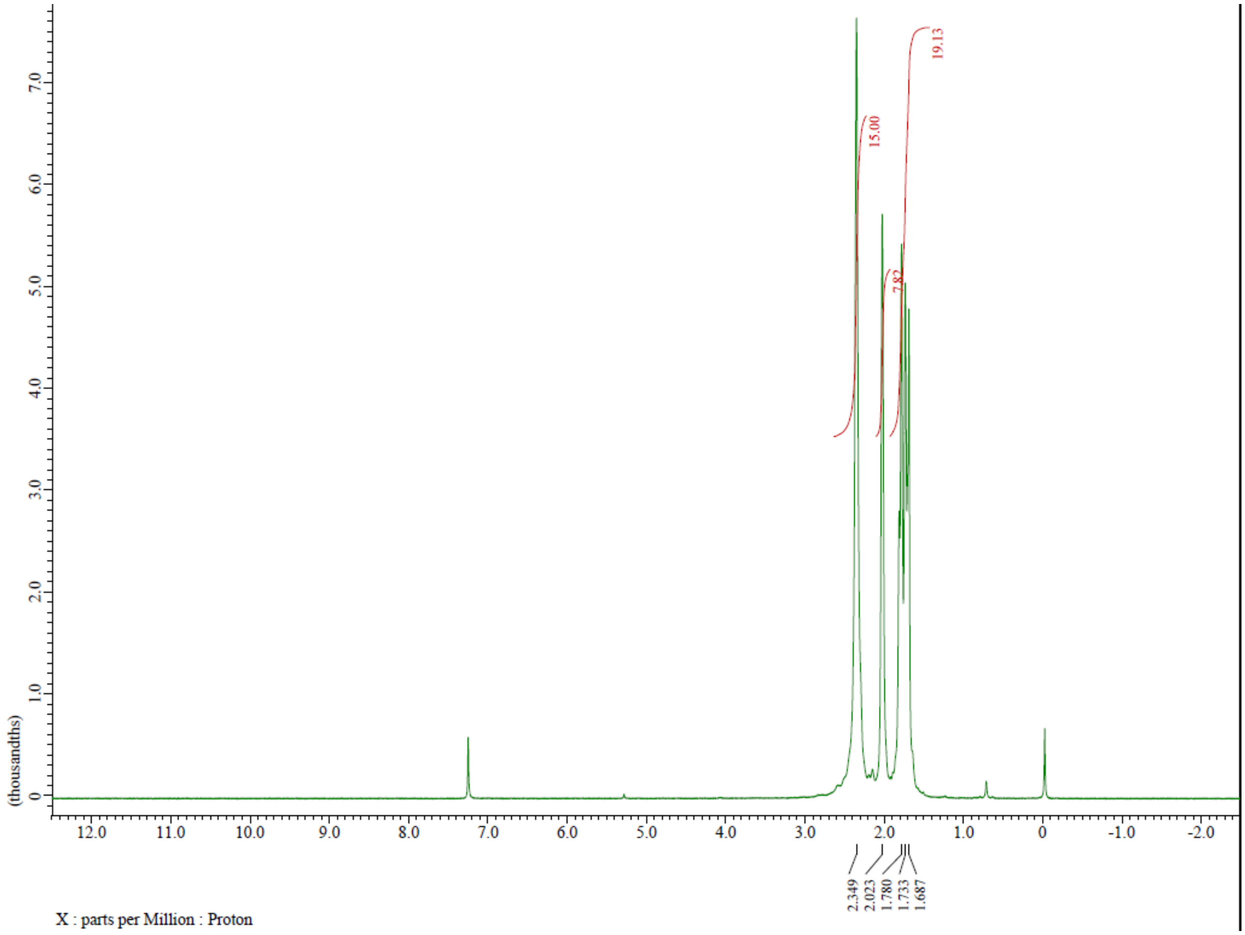

Supplementary Figure 4: ^{1}H NMR of **9**

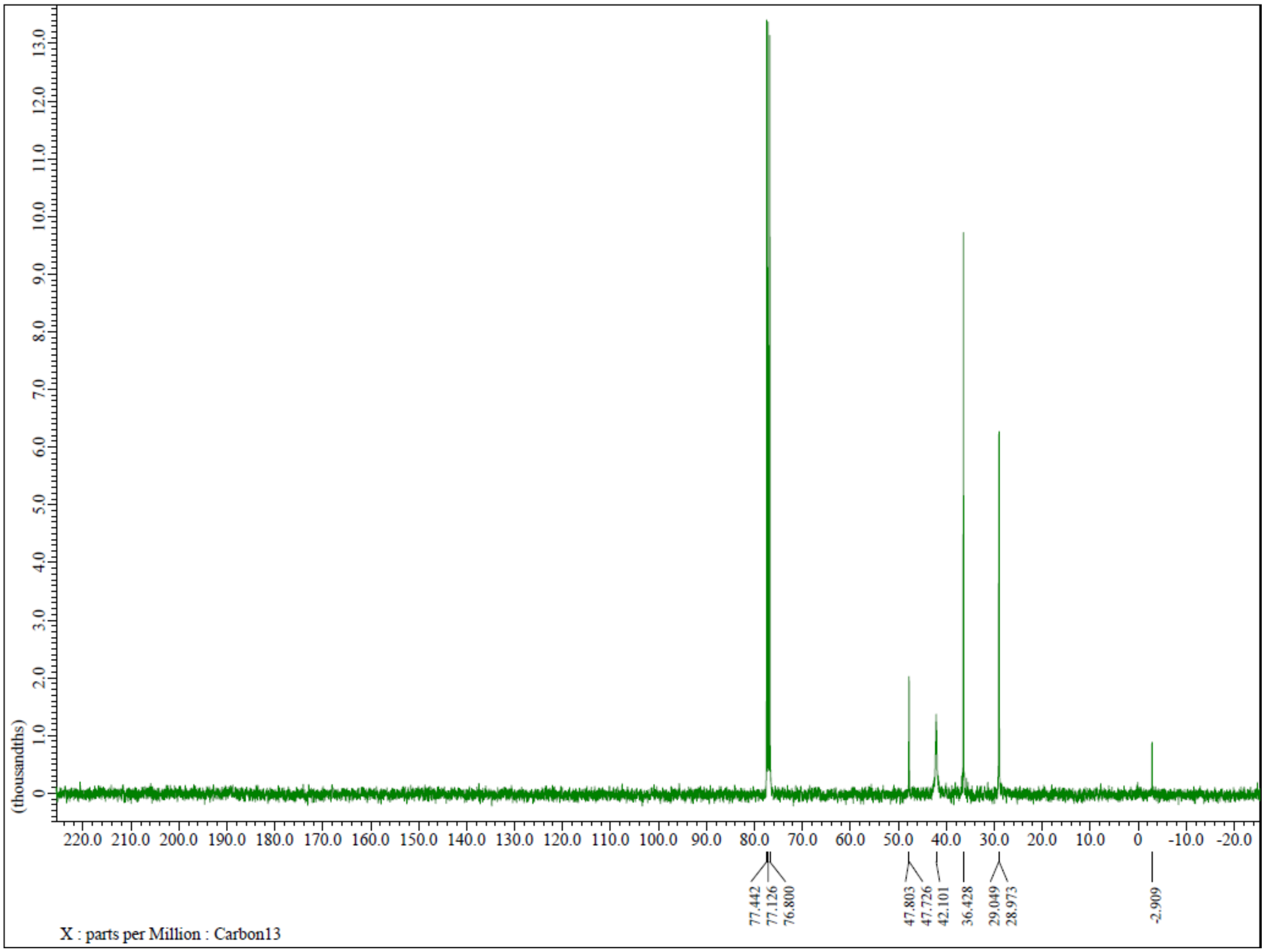

Supplementary Figure 5: ^{13}C NMR of **9**

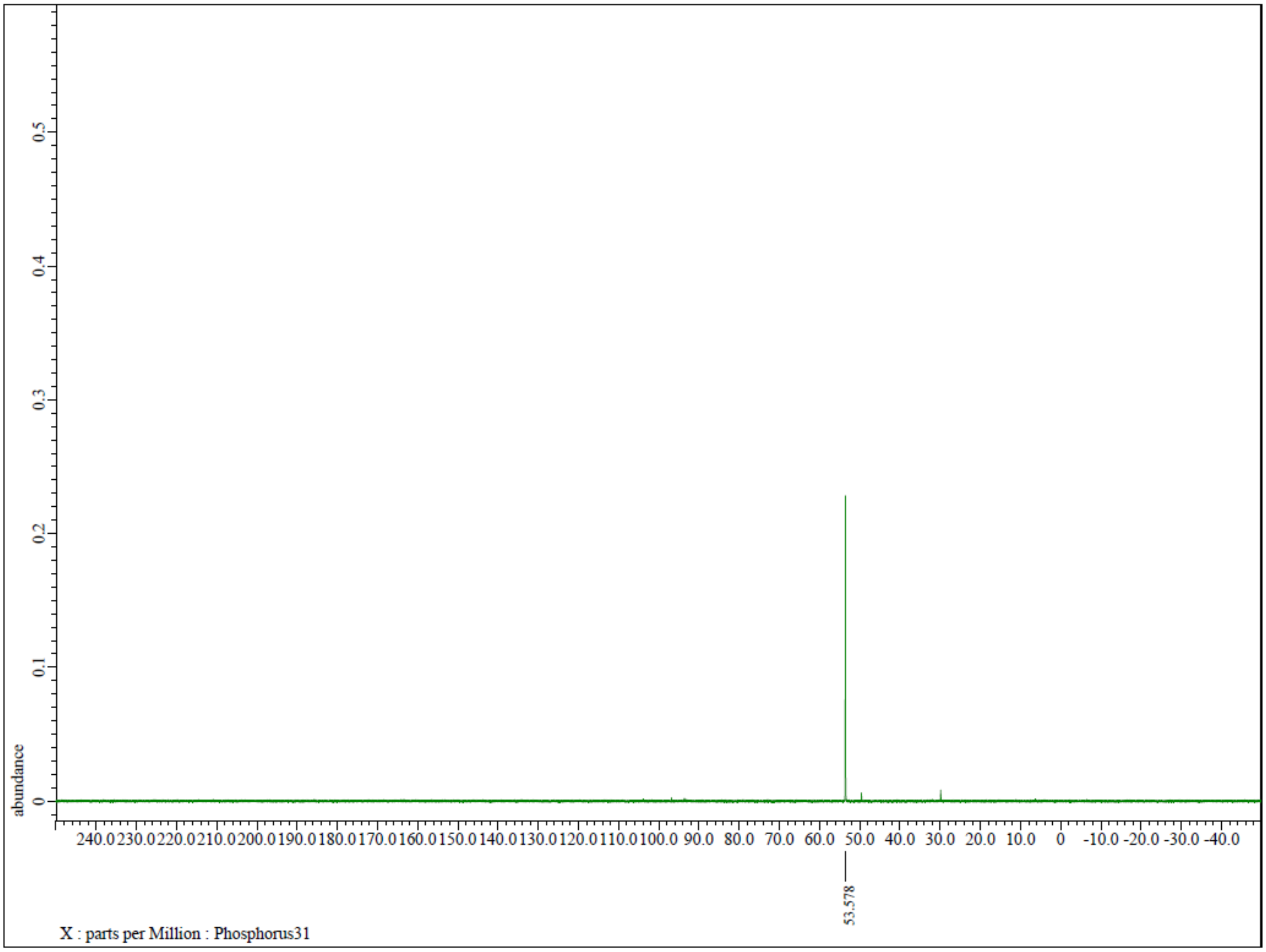

Supplementary Figure 6: ^{31}P NMR of **9**

Supplementary Tables

Crystallographic Data for [Ad_3PNiBr_3][Ad_3PH]

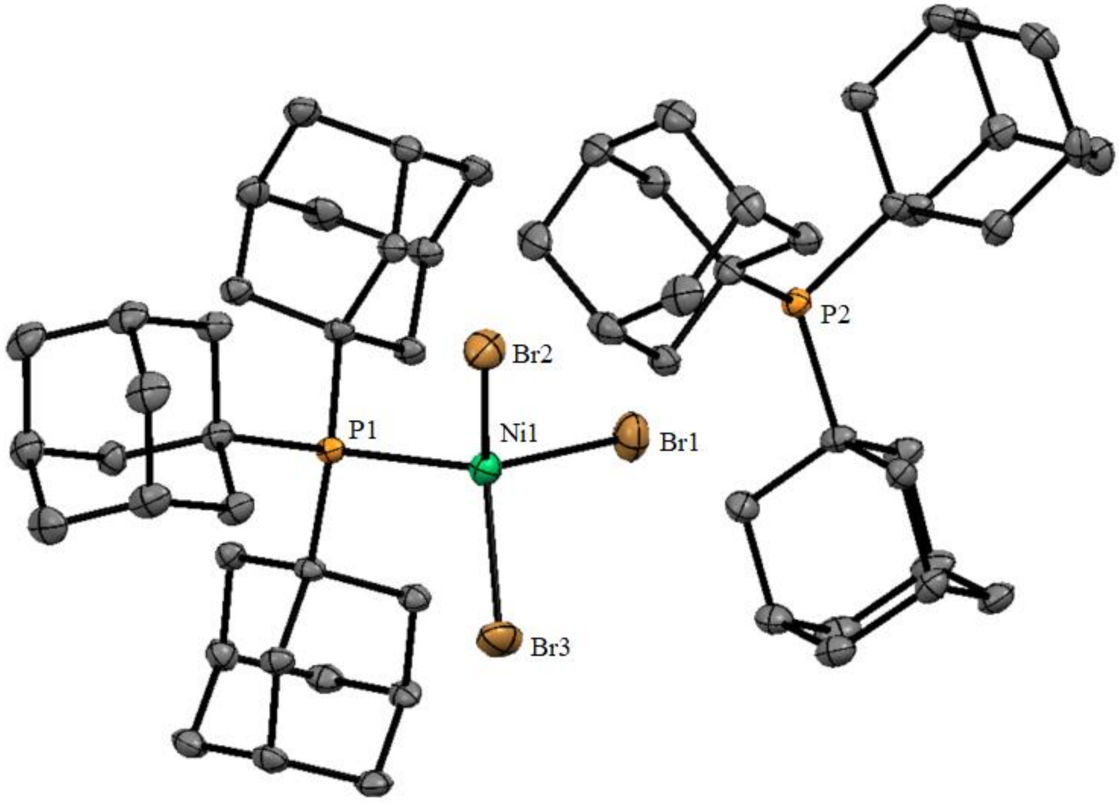

Supplementary Figure 7: ORTEP View (50% probability of ellipsoids) of **6** showing atom labeling. Hydrogens and chlorobenzene solvate omitted for clarity.

Supplementary Table 2: Crystal data and structure refinement for 6

Empirical formula	C66 H96 Br3 Cl Ni P2	
Formula weight	1285.25	
Temperature	123(2) K	
Wavelength	1.54178 Å	
Crystal size	0.08 x 0.03 x 0.005 mm^3	
Crystal system	Monoclinic	
Space group	P 21/c	
Unit cell dimensions	a = 16.1279(2) Å	α= 90°.
	b = 17.0766(2) Å	β= 96.906(1)°.
	c = 21.2287(3) Å	γ = 90°.
Volume	5804.17(13) $Å^3$	

Z	4
Density (calculated)	1.471 Mg/m^3
Absorption coefficient	4.146 mm^{-1}
F(000)	2688
Theta range for data collection	2.760 to 66.664°.
Index ranges	-18<=h<=19, -19<=k<=20, -25<=l<=24
Reflections collected	38022
Independent reflections	10191 [R(int) = 0.0492]
Completeness to theta = 66.664°	99.3 %
Absorption correction	Empirical
Max. and min. transmission	0.7528 and 0.6319
Refinement method	Full-matrix least-squares on F^2
Data / restraints / parameters	10191 / 322 / 724
Goodness-of-fit on F^2	1.000
Final R indices [I>2sigma(I)]	R1 = 0.0368, wR2 = 0.0886
R indices (all data)	R1 = 0.0512, wR2 = 0.0963
Largest diff. peak and hole	0.811 and -0.790 e.Å^{-3}

Supplementary Table 3: Homopolymerization of ethylene catalyzed by **6**

#	Solvent Volume	Catalyst loading	Pressure (PSI)	Time (min)	Temperature (°C)	TON x 10^{-4}	TOF x 10^{-5} h^{-1}	M_w x 10^{-3}	M_n x 10^{-3}	Đ	Tm (°C)
1	150 mL	2 µmol	200	30	25	6.2	1.2	1040	640	1.63	131
2	150 mL	1 µmol	200	30	25	9.6	1.9	1130	687	1.64	129.9
3	150 mL	0.5 µmol	200	30	25	18	3.6	1120	768	1.46	131.2
4	250 mL	0.5 µmol	200	30	25	18	3.7	975	749	1.30	133.2
5	150 mL	0.5 µmol	400	30	25	22	4.4	1360	793	1.72	133.2
6	150 mL	0.5 µmol	100	3.5	10	7.9	13	1330	944	1.41	132.4
7	150 mL	0.5 µmol	100	7.5	10	14	11	1820	1410	1.29	131.6
8	150 mL	0.5 µmol	100	15	10	22	8.9	2340	1680	1.39	131.8
9	150 mL	0.5 µmol	100	30	10	23	4.6	2270	1640	1.38	130.8
10	150 mL	0.5 µmol	200	3.5	10	17	28	1980	1450	1.37	131.8
11	150 mL	0.5 µmol	200	7.5	10	22	18	2030	1570	1.22	131.6
12	150 mL	0.5 µmol	200	15	10	26	10	2130	1650	1.29	131.1
13	150 mL	0.5 µmol	400	3.5	10	22	37	1900	1390	1.36	132.6
14[b]	150 mL	0.5 µmol	200	30	25	2.8	0.56	201	89	2.26	n.d.
15[c]	150 mL	1 µmol	200	30	25	5.0	1.0	1290	930	1.38	132.7
16[d]	150 mL	1 µmol	200	30	25	11	2.2	1130	696	1.62	129.9
17[3]	150 mL	1 µmol	200	30	25	12	2.3	1330	772	1.72	130.9
18	150 mL	0.5 µmol	200	15	25	20	7.9	1180	838	1.41	132.4
19	150 mL	0.5 µmol	200	15	50	11	4.4	600	411	1.46	n.d.
20	150 mL	0.5 µmol	200	15	75	2.1	0.85	239	171	1.40	n.d.
21[f]	150 mL	0.5 µmol	200	7.5	10	22	17	1410	1100	1.28	n.d.
22[g]	150 mL	0.5 µmol	200	15	25	0	0	----	----	----	----

Conditions: toluene, PMAO-IP 1750 eq, catalyst added as a CH_2Cl_2 solution, absolute molecular weight determined by GPC in 1,2,4-trichlorobenzene, Tm determined by DSC. b) $[tBu_3PNiBr_3]^-[tBu_3PH]^+$ catalyst, c) MMAO 1000 eq. d) PMAO-IP 1000 eq. e) PMAO-IP 2500 eq. f) **8** used as catalyst. g) 140 mL toluene, 10 mL EtOAc

Supplementary Table 4: Copolymerization of ethylene with α-olefins

#	Comonomer	Conc	Pressure (PSI)	Time (min)	Temp (°C)	TON x 10^{-5}	TOF x 10^{-5} h^{-1}	Mw x 10^{-3}	Mn x 10^{-3}	Đ	χ	Tm (°C)
1	1-hexene	0.25 M	200	30	25	2.1	4.2	546	335	1.63	2.7	106.4
2	1-hexene	0.50 M	200	30	25	2.3	4.6	452	293	1.54	5.1	91.0
3	1-hexene	1.0 M	200	30	25	2.4	4.7	400	272	1.47	5.6	85.3
4	1-hexene	0.25 M	400	30	25	2.5	4.9	523	344	.1.52	2.6	106.7
5	1-hexene	0.50 M	0	30	25	0	0	----	----	----	----	----
6	1-octadecene	0.25 M	200	3.5	20	2.2	38	535	374	1.43	1.9	n.d.
7	1-octadecene	0.25 M	200	15	20	2.2	8.6	503	373	1.35	1.8	n.d.
8	9D1OTBS[b]	0.25 M	200	15	15	2.3	9.3	1243	879	1.41	1.2[c]	n.d.
9	methyl acrylate	0.25 M	200	30	25	0	0	----	----	----	----	----

Conditions: toluene, PMAO-IP 1750 eq, catalyst added as a CH_2Cl_2 solution, absolute molecular weight determined by GPC in 1,2,4-trichlorobenzene, comonomer incorporation determined by ^{13}C NMR, Tm determined by DSC. b) tert-Butyldimethyl(dec-9-en-1-yloxy)silane. c) Incorporation determined by ^{1}H NMR analysis.

Supplementary Table 5: Control Experiments

#	Change from Standard Cond	Yield (mg)	TOF x 10^{-3} h^{-1}	Mw x 10^{-3}	Mn x 10^{-3}	Đ
1	[tBu_3PNiBr_3] [tBu_3PH] used as catalyst, no **6**	392	56	201	89	2.26
2	Add 10 eq Ad_3P	497	71	1610	1090	1.47
3	Ad_3P only (1 µmol), no **6**	0	0	----	----	----
4	$NiBr_2$*$3H_2O$ (0.5 µmol), no **6**	27	3.9	----	----	-----

Standard conditions: toluene 150 mL, catalyst **6** (0.5 µmol) added as CH_2Cl_2 solution, 30 min, 25 °C, 200 PSI, PMAO-IP 1750 eq.

Supplementary Figures

Sample DSC Data

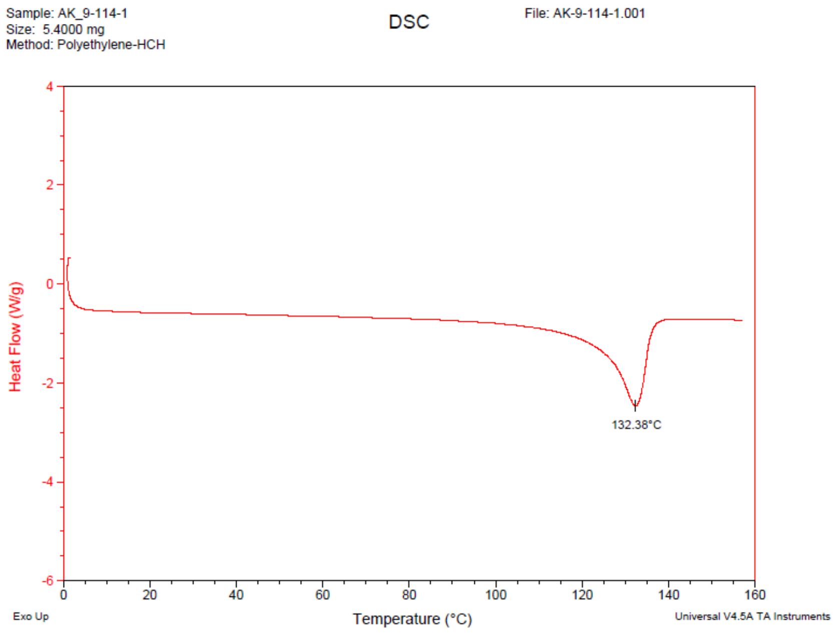

Supplementary Figure 8: DSC Measurements for Table 1, Entry 6.

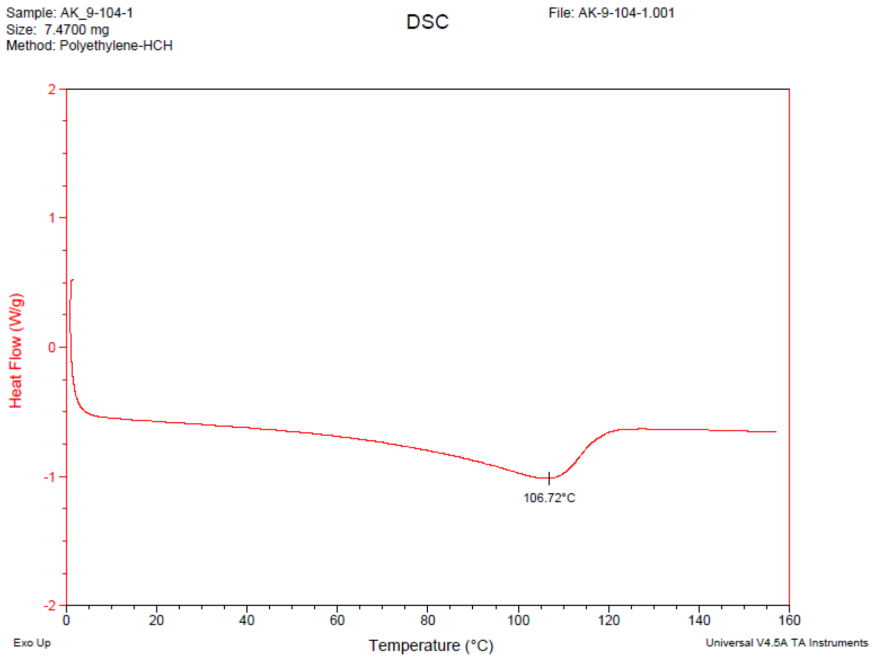

Supplementary Figure 9: DSC Measurements for Table 2, Entry 4.

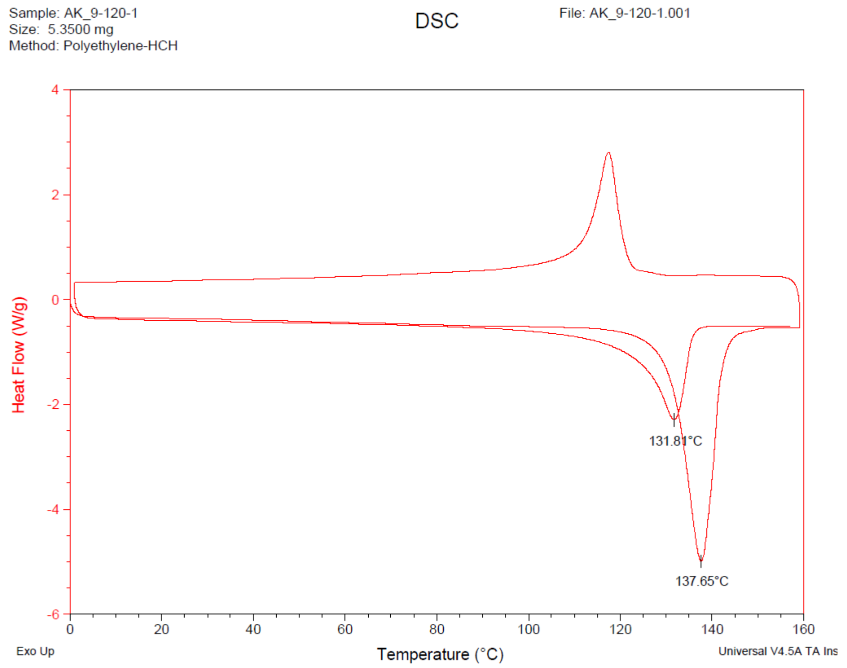

Supplementary Figure 10: Full DSC curve showing first and second melt for Table 1, Entry 8

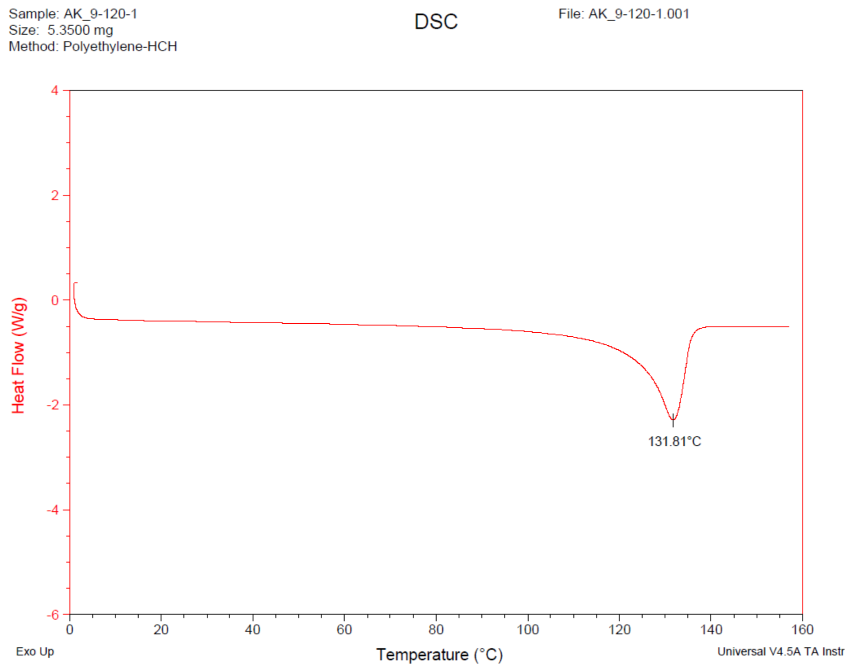

Supplementary Figure 11: Isolated second melt for Table 1, Entry 8

Sample GPC Data

ID	AK_9-114-1

Acq.	Feb 10, 2018 - 17:04:09

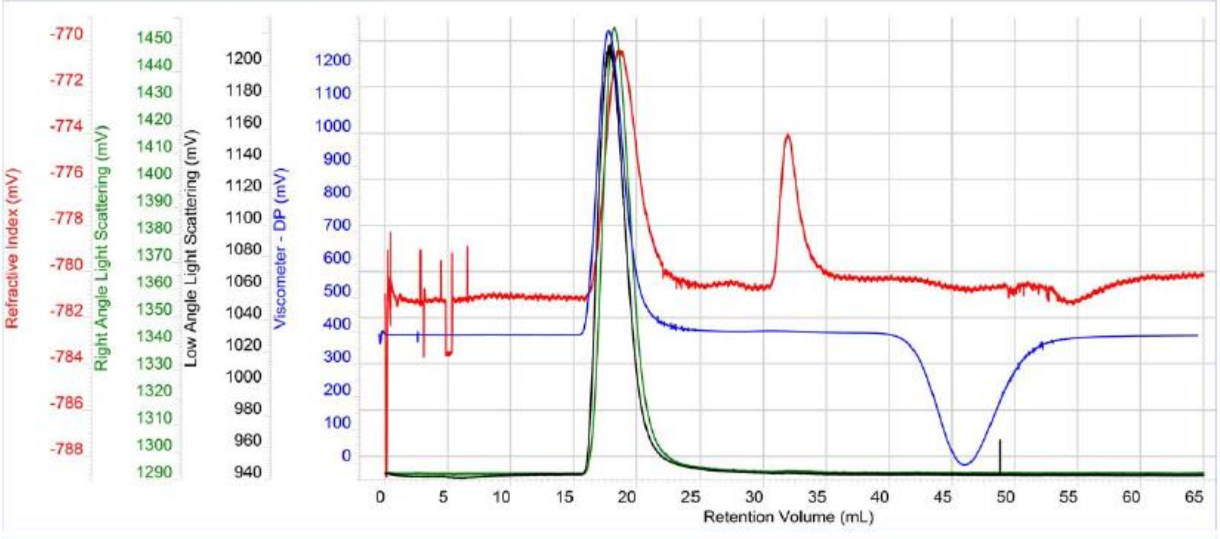

ID	AK_9-114-1
Mw	1.334 e 6
Mn	944,573
Mz	2.226 e 6
Rg	81.8336
Acq. Date	Feb 10, 2018 - 17:04:09
Inj Vol	200.0
Flow Rate	1.0000
Calculated	1.7376
Recovery	82.7450
dn/dc	0.1000
Solvent	TCB
Method	20180216_Calibration-0010.vc

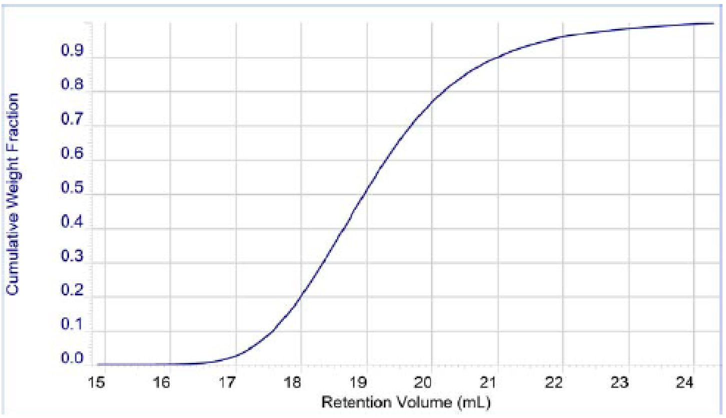

Supplementary Figure 12: GPC Measurements for Table 1, Entry 6.

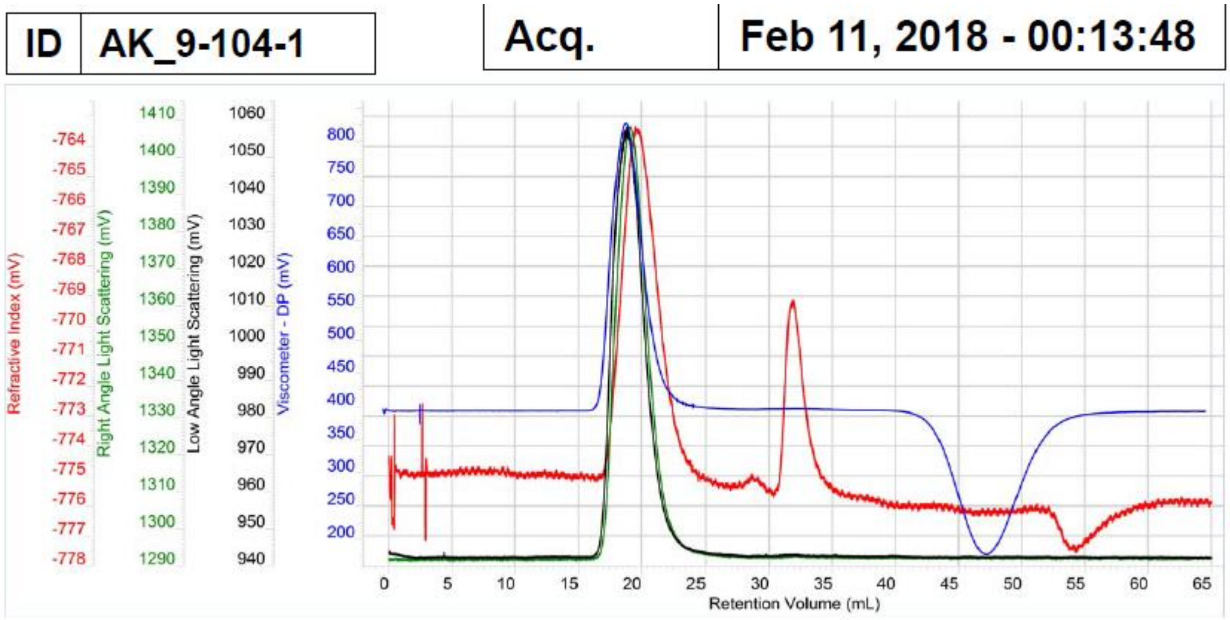

ID	AK_9-104-1
Mw	523,751
Mn	344,159
Mz	895,083
Rg	47.0346
Acq. Date	Feb 11, 2018 - 00:13:48
Inj Vol	200.0
Flow Rate	1.0000
Calculated	2.0578
Recovery	96.1595
dn/dc	0.1000
Solvent	TCB
Method	20180216_Calibration-0010.vc

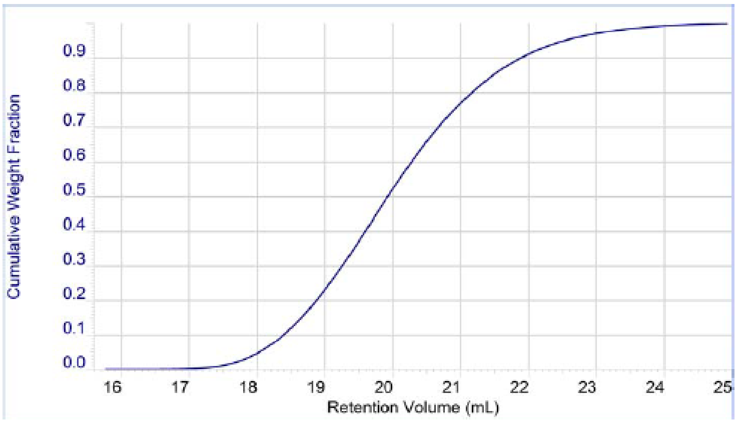

Supplementary Figure 13: GPC Measurements for Table 2, Entry 4.

Sample NMR Data

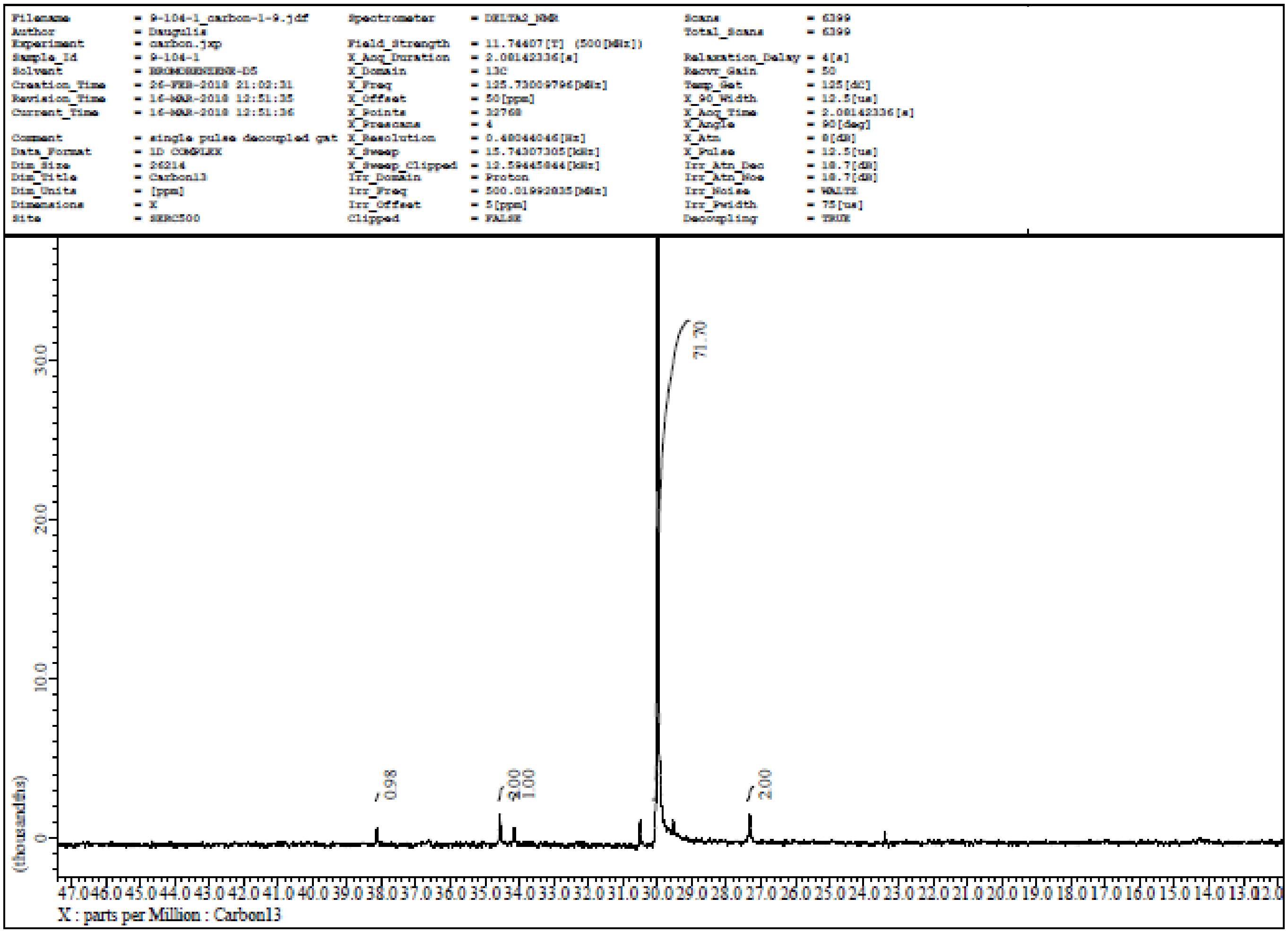

Supplementary Figure 14: ^{13}C NMR for Table 2, entry 4.

To calculate the incorporation, the following formula was used:

$[CH_2]$ = integration of the main backbone signal when the 1-hexene carbons have their integration normalized to 1.

Number of ethylene units per 1 hexene = $([CH_2]+5)/2$

$\chi\% = 100 * (1/([CH_2]+5)/2$

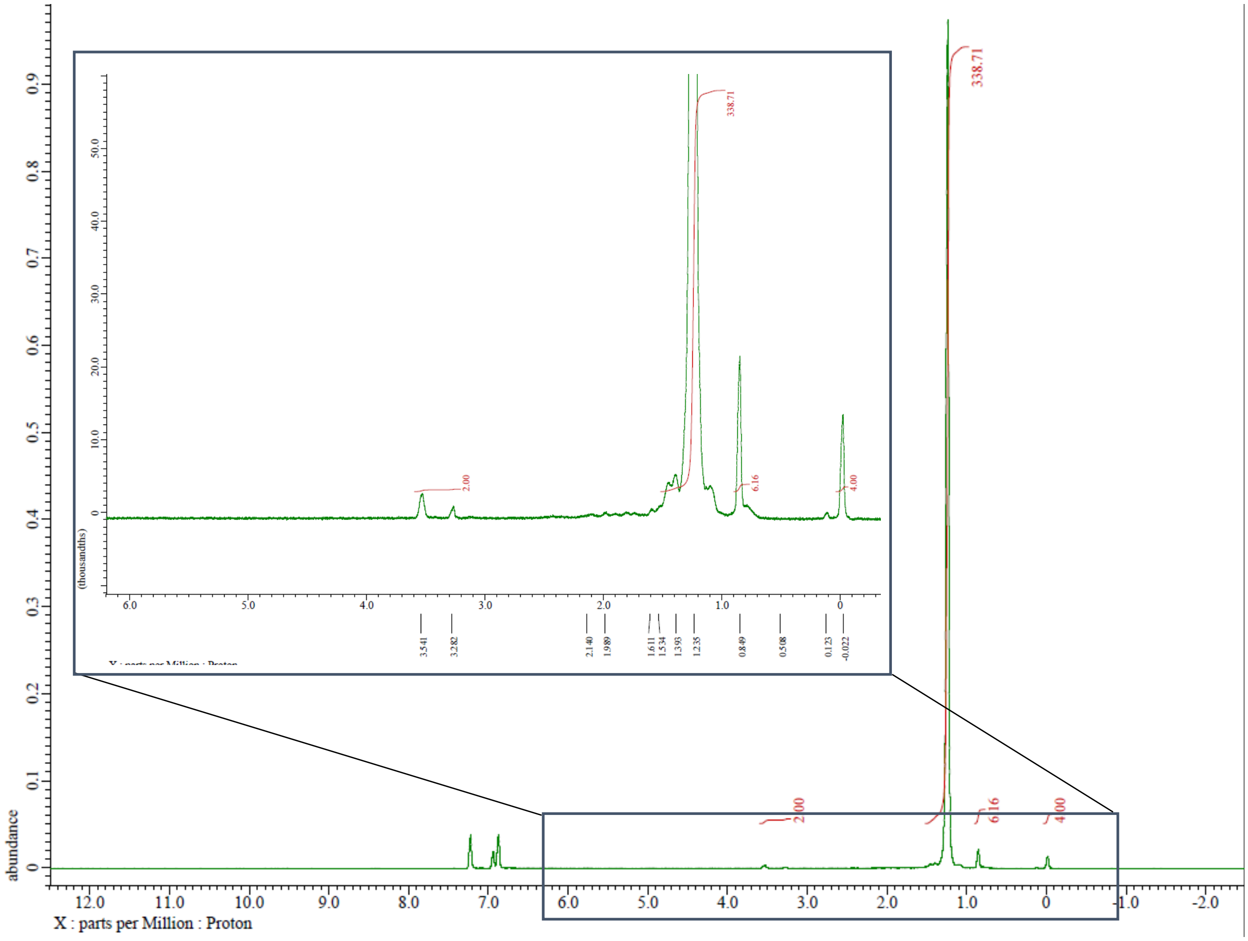

Supplementary Figure 15: ^{1}H NMR for Table 2, Entry 8.

To calculate the incorporation, the following formula was used:

[CH_2] = integration of the main backbone signal when the O-CH_2- signals (two from –OTBS and –OH end groups) from the comonomer has its integration normalized to 2. Hydroxyl groups originate from partial deprotection of the silyl moiety. In calculating the TOF for table 2, entry 8, it was assumed all groups have -OTBS end groups. This causes a slight underestimation of the TON.

Number of “ethylene” units per 1 comonomer = ([CH_2]-17)/4

$\chi\% = 100 * (1/([CH_2]-17)/4$

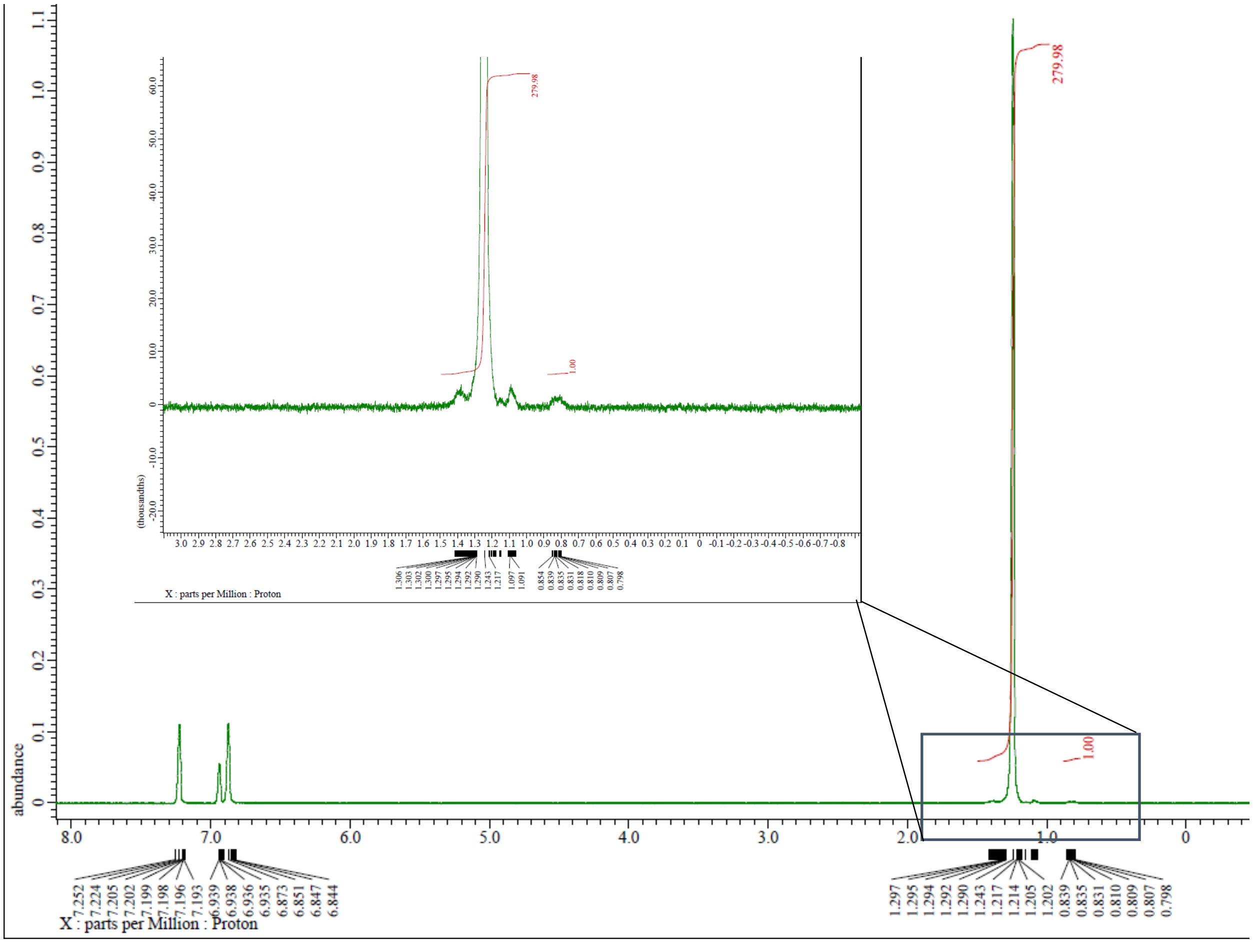

Supplementary Figure 16: ^{1}H NMR for Table 1, Entry 15

Supplementary References

1. Chen, L., Ren, P. & Carrow, B. P. Tri(1-adamantyl)phosphine: Expanding the Boundary of Electron-Releasing Character Available to Organophosphorus Compounds. *J. Am. Chem. Soc*. **138**, 6392-6395 (2016).
2. Balas, L. et al. Regiocontrolled syntheses of FAHFAs and LC-MS/MS differentiation of regioisomers. *Org. Biomol. Chem*. **14**, 9012-9020 (2016).
3. Alyea, E. C., Fey, G. T., Goel, R. M. Psuedotetrahedral Metal(II) Complexes of Tris-(t-Butyl) Phosphine. *J. Coord. Chem*. **5**, 143-152 (1976).
